# Data on plasma cortisol levels in nibbler fish *Girella punctata* reared under high-density conditions in either surface seawater or deep ocean water

**DOI:** 10.1016/j.dib.2023.109361

**Published:** 2023-07-04

**Authors:** Takahiro Ikari, Jun Hirayama, Muhammad Ahya Rafiuddin, Yukihiro Furusawa, Yoshiaki Tabuchi, Kazuki Watanabe, Atsuhiko Hattori, Ryotaro Kawashima, Kitaro Nakamura, Ajai K. Srivastav, Kenji Toyota, Hajime Matsubara, Nobuo Suzuki

**Affiliations:** a Noto Marine Laboratory, Institute of Nature and Environmental Technology, Kanazawa University, Ogi, Noto-cho, Ishikawa 927-0553, Japan; b Department of Clinical Engineering, Faculty of Health Sciences, Komatsu University, Komatsu, Ishikawa 923-0961, Japan; c Division of Health Sciences, Graduate School of Sustainable Systems Science, Komatsu University, Komatsu, Ishikawa 923-0961, Japan; d Noto Center for Fisheries Science and Technology, Kanazawa University, Ossaka, Noto-cho, Ishikawa 927-0552, Japan; e Department of Pharmaceutical Engineering, Faculty of Engineering, Toyama Prefectural University, Kurokawa, Toyama 939-0398, Japan; f Life Science Research Center, University of Toyama, Sugitani, Toyama 930-0194, Japan; g Department of Sport and Wellness, College of Sport and Wellness, Rikkyo University, Kitano, Niiza, Saitama 352-8558, Japan; h Department of Zoology, D.D.U. Gorakhpur University, Gorakhpur 273-009, India

**Keywords:** Marine teleost, Density stress, Stress-reducing effect, Aquaculture

## Abstract

Deep ocean water (DOW) is the water obtained from depth of >200 m below the surface of Earth's oceans and is characterized by rich nutrients and cleanliness [1,2]. We have recently reported that DOW suppresses the high-density-induced increase of plasma cortisol levels (i.e., a stress marker) in Japanese flounder (*Paralichthys olivaceus*) [1]. The current study aimed to examine whether the cortisol-reducing effect of DOW was observed in other marine organisms as well by comparing the plasma cortisol levels of nibbler fish *Girella punctata* reared under high-density conditions between surface seawater (SSW) and DOW. The nibbler fish were caught from Tsukumo Bay of Noto Peninsula (Ishikawa Prefecture, Japan). The DOW was obtained from seawater 320 m below the Noto Bay surface at a facility (Aquas Noto, Ishikawa Prefecture, Japan), whereas SSW was obtained from Tsukumo Bay (Noto Peninsula, Ishikawa Prefecture). The dissolved oxygen was maintained at approximately 7 mg/L in DOW as well as in SSW. Before they were transferred to the high-density condition, nibbler fish were acclimated in SSW at 20°C for 1 week at a mean density of 100 g/62.5 L. To expose them to the high-density stress, each of fish was kept at a density of 10 kg/m^3^ in a single aquarium (60 × 25 × 30 cm) containing either SSW or DOW (*n* = 8). Subsequently, the fish were reared with SSW or DOW for 10 days at 20°C ± 1°C under a 12:12-h light–dark cycle. A heparin containing syringe was used to obtain the blood samples from the caudal vessels of the fish anesthetized with a 0.04% 2-phenoxyethanol (FUJIFILM Wako Pure Chemical Corporation). The blood sampling was performed on days 0, 5, and 10 after rearing in the small aquaria. The plasma samples were prepared from the collected blood by centrifuging it at 5200 × g for 5 min and the cortisol concentrations were determined using an enzyme-linked immunosorbent assay (ELISA) kit (Cosmo Bio Co. Ltd., Tokyo, Japan) from those samples. The plasma cortisol concentration of nibbler fish reared in SSW on day 10 was significantly higher than that on day 0, whereas those reared in DOW did not show significant difference on the respective days. The current data contributes to the generalization of the cortisol-reducing effect of DOW on fish, which has been proposed in Japanese flounder [1]. These data could be used for developing and designing experiments to analyze the mechanisms underlying the cortisol-reducing effects by using small fish such as zebrafish, a well-established animal model.

**Specifications Table**

**Table:** 

Subject	Marine Biology
Specific subject area	Evaluation of the stress-reducing effect of ocean water on marine teleost
Type of data	Table Graph Figure
How the data were acquired	Enzyme-linked immunosorbent assay (ELISA)
Data format	Raw
Description of data collection	The nibbler fish *Girella punctata*, which had been maintained under a low-density condition with surface seawater (SSW), were exposed to a high-density condition. During stress exposure, the fish were reared in SSW or deep ocean water (DOW).
	Blood was obtained from the caudal vessels of the fish on days 0, 5, and 10 after exposure to high-density stress. The obtained blood samples were centrifuged at 5200 × g for 5 min to obtain the plasma samples. The cortisol concentrations in the prepared plasma samples were determined using an enzyme-linked immunosorbent assay.
Data source location	Noto Marine Laboratory, Institute of Nature and Environmental Technology, Kanazawa University Noto-cho, Ogi, Japan
Data accessibility	With the article
Related research article	T. Ikari, Y. Furusawa, Y. Tabuchi, Y. Maruyama, A. Hattori, Y. Kitani, K. Toyota, A. Nagami, J. Hirayama, K. Watanabe, A. Shigematsu, M.A. Rafiuddin, S. Ogiso, K. Fukushi, K. Kuroda, K. Hatano, T. Sekiguchi, R. Kawashima, A.K. Srivastav, T. Nishiuchi, A. Sakatoku, M.A. Yoshida, H. Matsubara, N. Suzuki, Kynurenine promotes Calcitonin secretion and reduces cortisol in the Japanese flounder Paralichthys olivaceus, Sci. Rep. 13(1) (2023) 8700. https://doi.org/10.1038/s41598-023-35222-4.

## Value of the Data

• Farmed fish are often reared at high densities, which has been thought to stress fish [2], [3], [4]. In aquaculture, measures to reduce stress should be considered to improve fish welfare [5]. Based on the current data and data from our recent study [1], using DOW for rearing marine organisms is expected to solve problems associated with stress induced by high densities.
• Researchers and fish farmers working on optimizing fish-farming conditions and methodologies to reduce stress induced by high densities of farmed fish can benefit from our data.
• The current data contributes to the generalization of the cortisol-reducing effects of DOW on fish. These data could be used to develop and design experiments to analyze the mechanisms underlying the cortisol-reducing effects using small fish such as zebrafish, a well-established animal model [6].
• Using small fish, a large-scale *in vivo* chemical screening via systematic exposure to chemical compounds can be performed in multi-well plates [7]. Accordingly, if it is confirmed that plasma cortisol levels can be used to evaluate stress induced by high densities in small fish such as zebrafish, density-stress-reducing chemical compounds will be screened using the plasma cortisol level as an indicator.

## 1 Objective

The seawater obtained from the depth of >200 m below the Earth's ocean surface is called DOW and has rich inorganic nutrients (nitrogen, phosphorus, and silicate) and clean water (with minimal to no bacterial activity and lower phytoplankton photosynthesis) [1,^2^,8]. The weight loss of squid (*Todarodes pacificus*) is reportedly suppressed when reared in DOW [9]. In addition to this positive DOW effect, our recent study revealed that in Japanese flounder (*Paralichthys olivaceus*) reared under high-density condition, the DOW-reared fish had lower plasma cortisol (i.e., a stress marker) levels than SSW-reared fish [1]. The current study provides data showing that DOW-reared nibbler fish had lower plasma cortisol levels under a high-density condition than SSW-reared nibbler fish. Accordingly, it helps generalize the cortisol-reducing effects of DOW on flounders.

## 2 Data Description

**Fig. 1:**
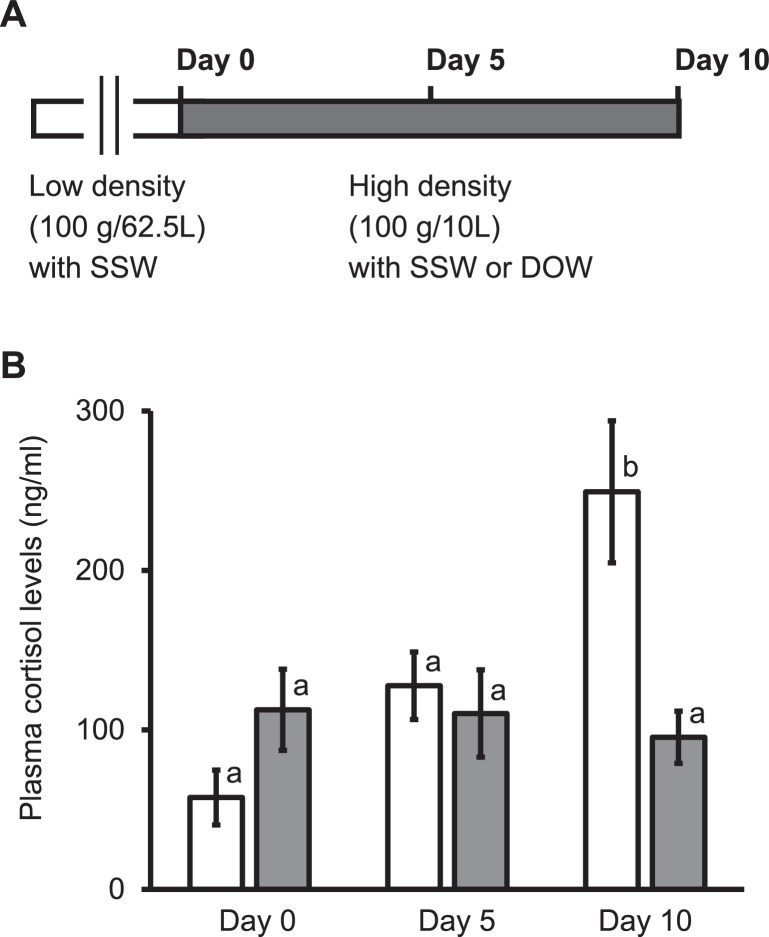
The plasma cortisol levels in nibbler fish on days 0, 5, and 10 after transferring them to a high-density condition with surface seawater (SSW) or deep ocean water (DOW).

(Fig. 1A) Schematic representation of the experimental setup for Fig. 1B. The nibbler fish were kept at a mean density of 100 g/62.5 L in SSW for 1 week (white bar). To expose them to a high-density condition, each of fish was then reared at a density of 100 g/10 L in a single aquarium (60 × 25 × 30 cm) containing either SSW or DOW (gray bar). The blood was collected on days 0, 5, and 10 after transferring the fish to a high-density condition. The white columns show plasma cortisol levels in nibbler fish transferred to a high-density condition with SSW (*n* = 8), whereas gray columns show plasma cortisol levels in nibbler fish transferred to a high-density condition with DOW (*n* = 8). The values are presented as means ± standard errors for eight independent experiments. The columns with different letters indicate significant differences (P  <  0.05). The plasma cortisol levels of nibbler fish maintained in SSW on day 10 were significantly higher than those on days 0 and 5, whereas those maintained in DOW showed no significant differences on days 0, 5, and 10. Additionally, the plasma cortisol levels on day 10 in the nibbler fish maintained in SSW were significantly higher than in those maintained in DOW.

Table 1 Raw data of the plasma cortisol concentrations of nibbler fish reared in SSW related to Fig. 1B. Of the eight nibbler fish maintained in SSW, the plasma cortisol levels on day 10 were higher than those on days 0 and 5.

**Table 1:** The plasma cortisol concentration (ng/ml) of nibbler fish reared at a density of 100 g/10 L in surface seawater for 0, 5, or 10 days.

Fish #	1	2	3	4	5	6	7	8
0 day	35.77	24.35	34.15	38.25	43.25	143.85	15.97	125.51
5 days	86.03	76.33	172.20	187.59	121.60	179.48	25.42	172.82
10 days	310.95	154.72	132.78	364.26	234.20	326.86	55.43	415.13

Table 2 Raw data of the plasma cortisol concentrations of nibbler fish reared in DOW related to Fig. 1B. Of the eight nibbler fish maintained in DOW, a common difference was not observed among the plasma cortisol levels on days 0, 5, and 10.

**Table 2:** The plasma cortisol concentration (ng/ml) of nibbler fish reared at a density of 100 g/10 L in deep ocean water for 0, 5, or 10 days.

Fish #	1	2	3	4	5	6	7	8
0 day	50.07	61.12	80.66	117.85	62.61	194.67	84.99	249.10
5 days	10.04	66.38	155.67	8.84	160.01	230.30	134.59	116.41
10 days	49.08	119.39	124.14	34.36	172.58	66.03	77.57	119.90

Table 3 Information on body weights of nibbler fish reared in SSW. The weight of all eight fish reared in SSW was found to be around 100 g.

**Table 3:** Body weights of nibbler fish reared in surface seawater.

Fish #	1	2	3	4	5	6	7	8	Means
Body weight (g)	89.2	108.7	86.3	116.4	93.6	107.8	92.7	108.9	100.5

Table 4 Information on body weights of nibbler fish reared in DOW. The weight of all eight fish reared in DOW was found to be around 100 g.

**Table 4:** Body weights of nibbler fish reared in deep ocean water.

Fish #	1	2	3	4	5	6	7	8	Means
Body weight (g)	93.6	115.1	98.2	100.0	83.0	102.2	101.8	112.2	100.8

## 3 Experimental Design, Materials and Methods

### 3.1 Animals

The nibbler fish *Girella punctata* were caught from Tsukumo Bay in the Noto Peninsula (Ishikawa Prefecture, Japan). Their average body weight was 100.6 ± 2.58 g. They were fed with artificial feed (4% body weight; Otohime, Marubeni Nisshin Feed Co., Ltd., Tokyo, Japan) once every morning. One-tenth of the water volume in each aquarium was replaced every day.

### 3.2 Rearing of nibbler fish in DOW or SSW

The DOW and SSW were pumped from Noto Bay (Aquas Noto, Ishikawa prefecture, Japan) and Tsukumo Bay (Noto Peninsula, Ishikawa Prefecture), respectively. The DOW used in this study was obtained from 320 m below the sea surface. In DOW and SSW, the dissolved oxygen concentration was maintained at approximately 7 mg/L. In DOW- or SSW-rearing systems, the wastes were filtered out in a filtration tank.

The nibbler fish were acclimated at the density of 1.6 kg/m^3^, after which they were maintained at a mean density of 100 g/62.5 L in the aquaria (200 × 100 × 65 cm) containing SSW for 1 week. To expose fish to the high-density condition, each of them was reared at a density of 10 kg/m^3^ in a single aquarium (60 × 25 × 30 cm) containing either SSW or DOW (*n* = 8). We considered that the density of 10 kg/m^3^ causes high-density stress based on the previous studies [1,3]. On days 0, 5, and 10 of exposure of fish to a high-density condition, the blood was drawn from caudal vessels of fish anesthetized with a 0.04% 2-phenoxyethanol (FUJIFILM Wako Pure Chemical Corporation). A heparinized syringe was used for blood sampling. To measure cortisol concentrations, the blood samples of fish were collected sequentially from the same tank. Subsequently, the obtained samples were centrifuged at 5200 × g for 5 min to obtain the plasma samples. They were then frozen and kept at −80°C until the plasma cortisol levels were measured as described in next section.

### 3.3 Plasma cortisol measurement

The plasma samples were mixed with a 5-fold volume of diethyl ether. The ether layer of the mixture was taken and evaporated with nitrogen gas. The dried samples were dissolved in an assay buffer (50 mM H_3_BO_3_, 0.2% bovine serum albumin, 0.01% thimerosal, and pH 7.8), and the plasma cortisol concentrations were determined using an ELISA kit (Cosmo Bio Co. Ltd., Tokyo, Japan).

### 3.4 Statistical analysis

The means of all groups were compared using analysis of variance. Multiple comparisons were evaluated using Tukey's honest significant difference tests. The *P*-values<0.05 were considered statistically significant. The statistical tests were performed using R version 4.2.3 software.

## Ethics Statements

### Statements on the ethical treatment of animals

This study was conducted strictly in accordance with the recommendations of the ethical guidelines of Kanazawa University. All the experimental protocols followed in this study were approved by the Animal Welfare Committee of Kanazawa University and were strictly in accordance with the ARRIVE guidelines 2.0 [10]. Maximum efforts were made to avoid causing pain and distress to experimental animals and special attention was paid to the use of anesthesia to alleviate discomfort.

## CRediT Author Statement

All authors contributed to the study conception and design. Material preparation (T.I., M.A.R., S.O., K.K., K.H., T.S., and R.K.), data collection (J.H., K.W., and A.S.), analysis (K.F., Y.K., H.M., A.N., T.N., and A.S.), and discussion (A.K.S, Y.F., Y.T., Y.M., A.H. M.A.Y., K.T., and N.S.) were performed. The first manuscript draft was written by N.S., Y.F., T.I., and J.H., and all authors commented on previous versions of the manuscript. All authors read and approved the final manuscript.

## Declaration of Competing Interest

The authors declare that they have no known competing financial interests or personal relationships that could have appeared to influence the work reported in this paper.

## Data Availability

Data on plasma cortisol levels in nibbler fish *Girella punctata* reared under high-density conditions and Information on body weights of nibbler fish used in this study (Original data) (Mendeley Data).

## References

[1] Ikari T., Furusawa Y., Tabuchi Y., Maruyama Y., Hattori A., Kitani Y., Toyota K., Nagami A., Hirayama J., Watanabe K., Shigematsu A., Rafiuddin M.A., Ogiso S., Fukushi K., Kuroda K., Hatano K., Sekiguchi T., Kawashima R., Srivastav A.K., Nishiuchi T., Sakatoku A., Yoshida M.A., Matsubara H., Suzuki N. (2023). Kynurenine promotes Calcitonin secretion and reduces cortisol in the Japanese flounder Paralichthys olivaceus. Sci. Rep..

[2] Conte F.S. (2004). Stress and the welfare of cultured fish. Appl. Anim. Behav. Sci..

[3] Zahedi S., Akbarzadeh A., Mehrzad J., Noori A., Harsij M. (2019). Effect of stocking density on growth performance, plasma biochemistry and muscle gene expression in rainbow trout (Oncorhynchus mykiss). Aquaculture.

[4] Gornati R., Papis E., Rimoldi S., Terova G., Saroglia M., Bernardini G. (2004). Rearing density influences the expression of stress-related genes in sea bass (Dicentrarchus labrax, L.). Gene.

[5] Raposo de Magalhães C., Schrama D., Farinha A.P., Revets D., Kuehn A., Planchon S., Rodrigues P.M., Cerqueira M. (2020). Protein changes as robust signatures of fish chronic stress: a proteomics approach to fish welfare research. BMC Genomics.

[6] Adhish M., Manjubala I. (2023). Effectiveness of zebrafish models in understanding human diseases-a review of models. Heliyon.

[7] Lam P.Y., Peterson R.T. (2019). Developing zebrafish disease models for in vivo small molecule screens. Curr. Opin. Chem. Biol..

[8] Mohd Nani S.Z., Majid F.A., Jaafar A.B., Mahdzir A., Musa M.N. (2016). Potential health benefits of deep sea water: a review. Evid. Complement. Alternat. Med..

[9] Hatano K., Yoshida M.A., Hirayama J., Kitani Y., Hattori A., Ogiso S., Watabe Y., Sekiguchi T., Tabuchi Y., Urata M., Matsumoto K., Sakatoku A., Srivastav A.K., Toyota K., Matsubara H., Suzuki N. (2023). Deep ocean water alters the cholesterol and mineral metabolism of squid Todarodes pacificus and suppresses its weight loss. Sci. Rep..

[10] Percie du Sert N., Ahluwalia A., Alam S., Avey M.T., Baker M., Browne W.J., Clark A., Cuthill I.C., Dirnagl U., Emerson M., Garner P., Holgate S.T., Howells D.W., Hurst V., Karp N.A., Lazic S.E., Lidster K., MacCallum C.J., Macleod M., Pearl E.J., Petersen O.H., Rawle F., Reynolds P., Rooney K., Sena E.S., Silberberg S.D., Steckler T., Würbel H. (2020). Reporting animal research: Explanation and elaboration for the ARRIVE guidelines 2.0. PLoS Biol..

